# Elucidating the intramolecular contrast in the STM images of 2,4,6-tris(4′,4′′,4′′′-trimethylphenyl)-1,3,5-triazine molecules recorded at room-temperature and at the liquid-solid interface

**DOI:** 10.1039/c9ra09681g

**Published:** 2020-02-05

**Authors:** Fabien Silly

**Affiliations:** TITANS, SPEC, CEA, CNRS, Université Paris-Saclay CEA Saclay F-91191 Gif sur Yvette France fabien.silly@cea.fr +33169088446 +33169088019

## Abstract

Star-shaped 2,4,6-tris(4′,4′′,4′′′-trimethylphenyl)-1,3,5-triazine molecules self-assemble at the solid–liquid interface into a compact hexagonal nanoarchitecture on graphite. High resolution scanning tunneling microscopy (STM) images of the molecules reveal intramolecular features. Comparison of the experimental data with calculated molecular charge density contours shows that the molecular features in the STM images correspond to molecular LUMO+2.

## Abstract 2

Intramolecular contrast in the STM images of 2,4,6-tris(4′,4′′,4′′′-trimethylphenyl)-1,3,5-triazine molecules recorded at room-temperature and at the liquid–solid interface.

## Graphical abstract

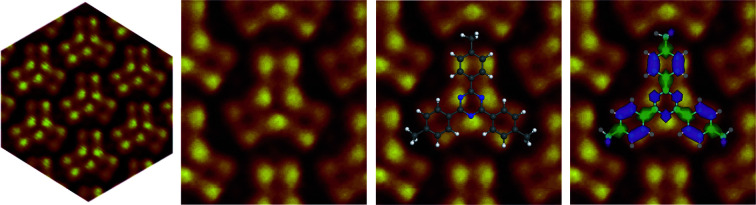

## 1 Introduction

Engineering novel two-dimensional (2D) organic nanoarchitectures is the focus of intense research interest^1–19^ for developing new materials for applications in nanotechnology, photovoltaics, spintronics and catalysis. Star-shaped molecules are promising building blocks to realize self-assembled structures. Star-shaped molecules usually constitute a central ring surrounded with three organic straight arms.^20–43^ High resolution imaging techniques are required not only to elucidate molecular assembly but also assess the electronic properties of individual molecules in the organic structure.

Recent technical developments have been perfected to improve the resolution of scanning probe microscopy (SPM) to separate surface topographic from electronic contribution in the SPM images of 2D nanostructures. Xe-functionalized and CO, H_2_ and D_2_ functionalized tips have been used to improve the resolution of non-contact atomic force microscopy (nc-AFM) in vacuum at low temperature.^44–62^ NaCl functionalised STM tip has been used to enhanced intramolecular features in organic and hybrid nanoarchitectures on a metal surface at room temperature in vacuum.^63^ It was thus possible to reveal that the intramolecular features correspond to the lowest unoccupied molecular orbitals (LUMO). SPM tips can hardly be functionalized at the solid–liquid interface at room temperature, but intramolecular features in organic layers have been sometimes reported.^64,65^ However these features are usually not assigned to the electronic or topographic properties of the molecule.

In this paper we investigate the self-assembly of 2,4,6-tris(4′,4′′,4′′′-trimethylphenyl)-1,3,5-triazine molecules at the 1-phenyloctane/graphite interface. This star-shaped molecule self-assembles into a compact nanoarchitecture on graphite at the solid–liquid interface. STM at room temperature reveals intramolecular details, corresponding to one of the molecular LUMOs states.

## 2 Experimental

Nearly saturated solutions of 2,4,6-tris(4′,4′′,4′′′-trimethylphenyl)-1,3,5-triazine (Aldrich) in 1-phenyloctane (Aldrich) were prepared. A droplet of the solution was then deposited on a highly oriented pyrolytic graphite substrate (HOPG). STM imaging of the samples was performed at the liquid–solid interface using a Pico-SPM (Molecular Imaging, Agilent Technology) scanning tunneling microscope. The surfaces were imaged using STM 1 h after molecular deposition. Cut Pt/Ir tips were used to obtain constant current images at room temperature with a bias voltage applied to the sample. STM images were processed and analyzed using the application FabViewer.^66^

## 3 Results

**Fig. 1:**
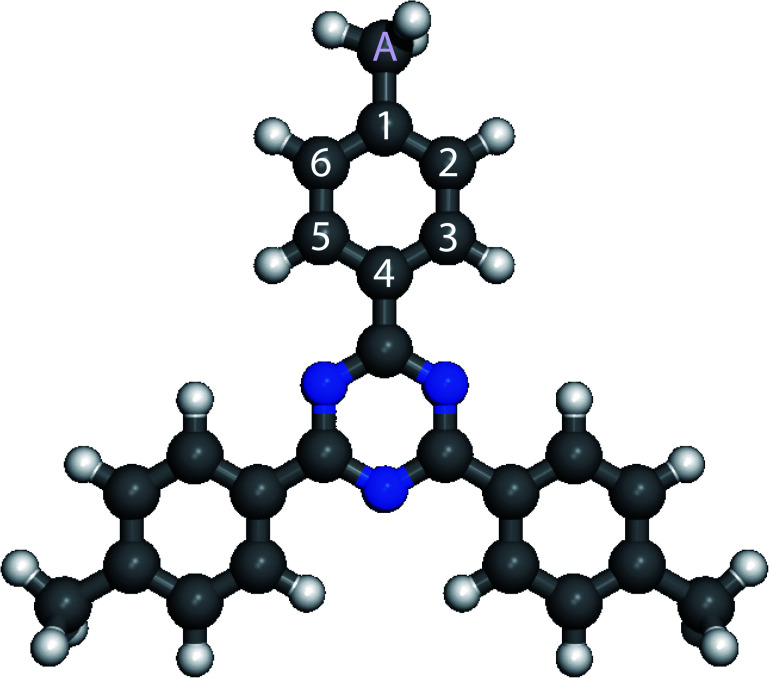
Scheme of 2,4,6-tris(4′,4′′,4′′′-trimethylphenyl)-1,3,5-triazine (C_24_H_21_N_3_) molecule. Carbon atoms are gray, nitrogen atoms blue, hydrogen atoms white, respectively. The six carbon atoms of the peripheral phenyl ring are labeled 1–6 and the carbon of the methyl group is labeled A.

The 2,4,6-tris(4′,4′′,4′′′-trimethylphenyl)-1,3,5-triazine molecule is 3-fold symmetry molecule. Its chemical structure is presented in Fig. 1. The skeleton of this star-shaped molecule consists of a central 1,3,5-triazine ring. This ring is connected to three peripheral benzene rings. Each benzene ring has one methyl group at its extremity. The carbon atom of the methyl group is labeled A, whereas the carbon atoms of the peripheral phenyl ring are labeled 1–6 in Fig. 1.

**Fig. 2:**
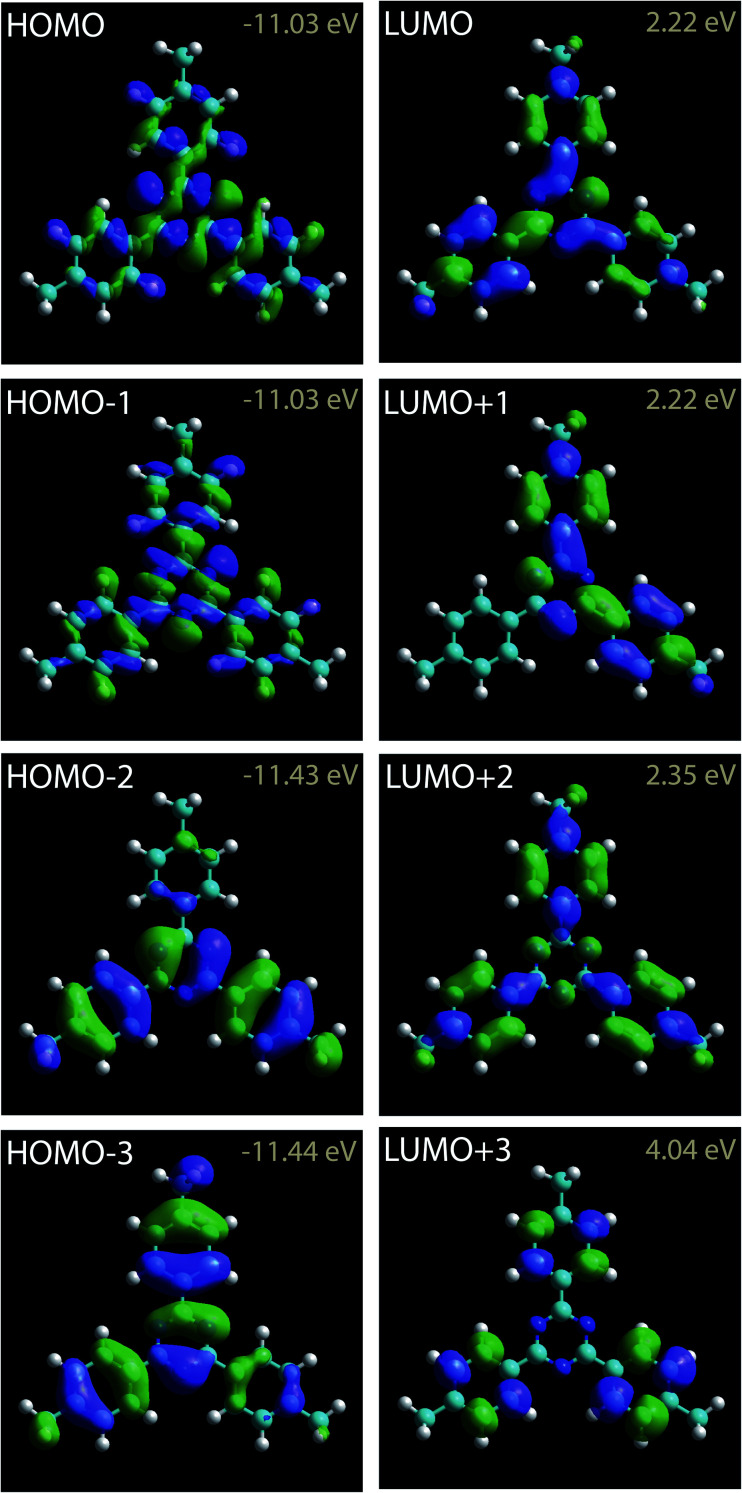
Charge density contours of the 2,4,6-tris(4′,4′′,4′′′-trimethylphenyl)-1,3,5-triazine LUMOs and HOMOs.

The calculated charge density contours of molecular four first lowest unoccupied molecular orbitals (LUMOs) and highest occupied molecular orbitals (HOMOs) for a free standing molecule are presented in the Fig. 2. The LUMOs and HOMOs contours were calculated using complete neglect of differential overlap (CNDO) semi empirical method.^67,68^

**Fig. 3:**
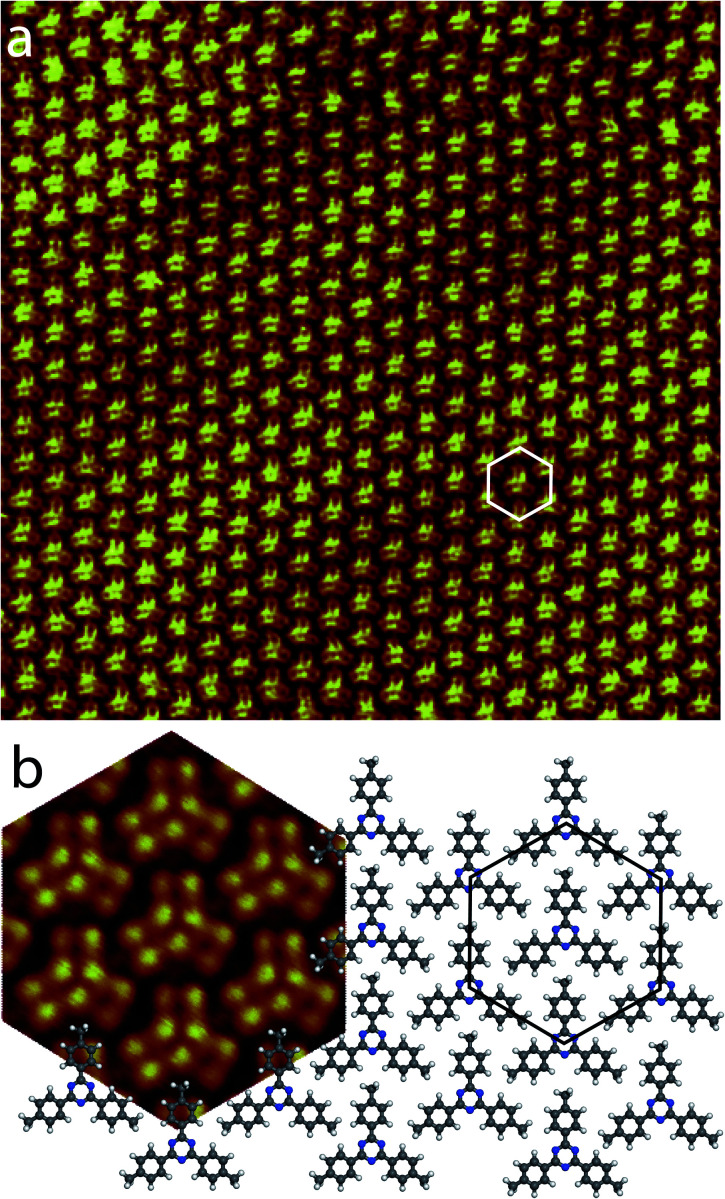
(a) Large scale STM image of the 2,4,6-tris(4′,4′′,4′′′-trimethylphenyl)-1,3,5-triazine self-assembled close-packed network at the 1-phenyloctane/graphite interface, 30 × 30 nm^2^, *V*_s_ = +0.27 V, *I*_*t*_ = 9 pA. The hexagonal unit cell (white) is superimposed to the STM image. (b) Model of the molecular arrangement. The unit cell is represented by a black hexagon. As guide for the eyes, a high resolution STM image of the hexagonal arrangement has been superimposed to the model (left).

Fig. 3a presents a large scale STM image of the graphite surface after deposition of a droplet of 2,4,6-tris(4′,4′′,4′′′-trimethylphenyl)-1,3,5-triazine molecules in 1-phenyloctane. STM shows that the molecules self-assemble into a hexagonal compact two-dimensional nanoarchitecture.^69^ This structure has a 1.4 nm hexagonal unit cell constant. The model of the molecular arrangement is presented in Fig. 3b. In this arrangement the each molecular phenyl group is pointing towards a nitrogen atom of a neighboring molecule.

**Fig. 4:**
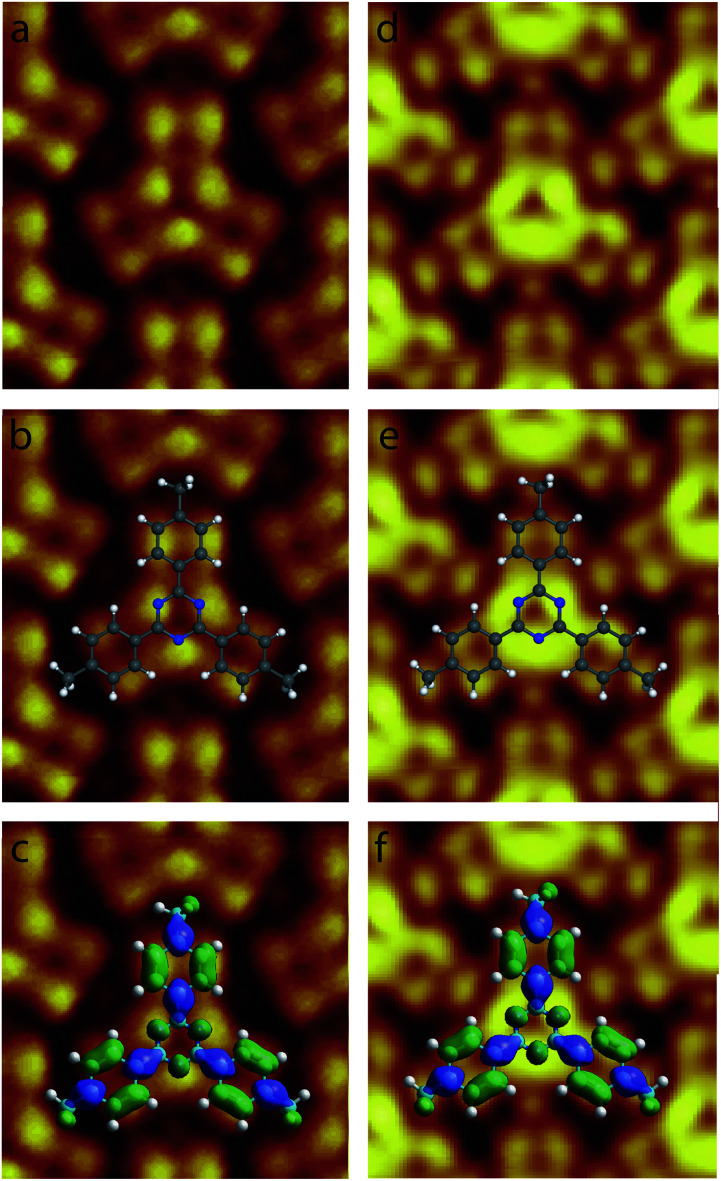
High resolution STM images of single the 2,4,6-tris(4′,4′′,4′′′-trimethylphenyl)-1,3,5-triazine molecules in the organic network, (a–c) 2 × 2 nm^2^*V*_s_ = +0.30 V, *I*_*t*_ = 9 pA, (d–f) 2 × 2 nm^2^, *V*_s_ = +0.27 V, *I*_*t*_ = 9 pA. The model of molecular skeleton with (c and f) and without (b and e) the calculated charge density contour of the LUMO+2 has been superimposed to the STM images.

High resolution STM images of the 2,4,6-tris(4′,4′′,4′′′-trimethylphenyl)-1,3,5-triazine molecule recorded for high resolution STM images were recorded at *V*_*t*_ ∼0.3 V, are presented in Fig. 4. The STM image in Fig. 4a has been recorded at *V*_s_ = +0.30 V, whereas the STM in Fig. 4d has been recorded at *V*_s_ = +0.27 V. The two STM images are revealing intramolecular details. As a guide for the eyes, molecular scheme has been superimposed to the two STM images in Fig. 4b and e, respectively. The two images in Fig. 4a and d show that the center ring of the molecule appears as three bright spots in the STM images. Theses bright spots correspond to the position of the nitrogen atoms in the central molecular triazine ring, Fig. 4b and e. In contrast with the central triazine ring, the three peripheral molecular benzene rings appear as two parallel capsule-shape bright features in the STM images, Fig. 4a and d. The bright capsule-shape features correspond to the paired carbon atoms labeled 2,3 and 5,6 in Fig. 1. The STM images are also revealing that subtle variation of tunneling bias drastically modify the contrast of intermolecular features. At *V*_s_ = +0.30 V, the intensity of the bright features in the peripheral benzene rings and the central triazine ring is similar in the STM images, Fig. 4a. In comparison the intensity of the bright features in the central triazine ring is higher than the one in the peripheral benzene rings at *V*_s_ = +0.27 V, Fig. 4d. The carbon atoms (labeled A in Fig. 1) of the molecular methyl-groups appear darker than the labelled 1,3,5,6 carbon atoms of the neighboring benzene ring at *V*_s_ = +0.30 V, Fig. 4a. At *V*_s_ = +0.30 V, the carbon atoms (A) of the molecular methyl-groups appear as bright as the labelled 1,3,5,6 carbon atoms of the neighboring benzene ring, Fig. 4d. The contrast of the central triazine ring is also changing with tunneling bias. At *V*_s_ = +0.30 V, the nitrogen atoms appear as bright as the side 1,3,5,6 carbon atoms of the benzene ring, whereas at *V*_s_ = +0.27 V they appear brighter.

## 4 Discussion

2,4,6-Tris(4′,4′′,4′′′-trimethylphenyl)-1,3,5-triazine molecules self-assemble into a compact arrangement on graphite. Scanning tunneling microscopy surprisingly reveals intramolecular details, despite the measurements have been performed at room temperature and at the solid–liquid interface. STM images recorded in the constant-current mode result from the convolution between the surface topography and the variation of surface integrated density of states. Very low temperature and vacuum conditions are usually required to acquire STM images with consistent submolecular resolution. Atomic details in molecular skeleton have been observed at the solid/liquid interface at room temperature^70^ but these data are rarely concretely compared to the molecular structure and molecular density of states. The experimental STM images in Fig. 4 reveal specific intramolecular features. The molecular central ring appears as three bright spots. The superimposition of molecular scheme to the STM images shows that the bright spot location corresponds to the position of molecular nitrogen atoms, Fig. 4b and e. The covalent bonds between the external carbon atoms of the peripheral molecular phenyl rings (covalent bonds between the carbon atoms labeled 2,3 and 5,6 in Fig. 1) also appear bright in the STM images. In comparison molecular methyl groups appear as round features in the STM images. The intensity of these features is strongly depending of the tunneling bias. At *V*_s_ = +0.30 V, their intensity is low and these features are darker than the other ones in the STM image, Fig. 4a, whereas they are as bright as the phenyl ring features at *V*_s_ = +0.27 V, Fig. 4d. Comparison of the intramolecular features with the calculated molecular charge density contour (presented in Fig. 2) reveals that the structure observed in the STM images corresponds to the molecular LUMO+2, as highlighted in Fig. 4c and f. It should be noticed that the molecular skeleton is flat except the methyl groups. The molecular methyl groups are therefore increasing the molecule-surface separation, which induces an electronic decoupling of the molecule from the surface. This may be at the origin of the appearance of the LUMO+2 in the STM images.

## 5 Conclusion

To summarize, scanning tunneling microscopy showed that star-shaped 2,4,6-tris(4′,4′′,4′′′-trimethylphenyl)-1,3,5-triazine molecules self-assemble into a close-packed nanoarchitecture on graphite at the liquid–solid interface. Intramolecular features are observed in the STM images at room temperature. Comparison of experimental molecular images with calculated molecular charge density contours reveals that molecular LUMO+2 is observed in the STM images.

## Conflicts of interest

There are no conflicts to declare.

## Supplementary Material
